# Well-Aware’ QI Project: Well-Being Support Awareness for Specialty Resident Doctors in Kent

**DOI:** 10.1192/bjo.2025.10451

**Published:** 2025-06-20

**Authors:** Suresh Thapaliya, Adeola Adeyemi, Eric Barratt, Rachel Daly

**Affiliations:** 1 Kent and Medway NHS and Social Care Partnership Trust, Canterbury, United Kingdom; 2 Kent and Medway NHS and Social Care Partnership Trust, Maidstone, United Kingdom; 3 Kent and Medway NHS and Social Care Partnership Trust, Dartford, United Kingdom

## Abstract

**Aims:** Doctors in training can be at high pressure to balance the service needs with their own training needs, thus leading to a risk of burnout. Hence, supporting their wellbeing is of paramount importance to optimize their training experience. This Quality Improvement (QI) project led to an improvement in well-being support for specialty trainee psychiatrists, training in Kent, United Kingdom.

**Methods:** This project employed a baseline survey among the specialty trainee psychiatrists training in Kent in Jan/Feb 2024 with support from the trusts’ medical education department and the wellbeing team. The survey questionnaire was modified from the NHS staff wellbeing survey. It utilized a Likert scale (1 to 5) to explore several aspects of awareness and access to available wellbeing resources. Additionally, open questions were included to collect views of the the resident doctors regarding wellbeing support.

Following the outcome of the baseline survey, the suggestions were implemented by the medical education department over a year and a follow-up survey was conducted to assess the impact of the changes. This project was registered with the QI department of the trust.

**Results:** Fifteen specialty trainee doctors participated in the baseline survey. The survey identified several gaps in the participants’ awareness about wellbeing resources such as; lack of information about the resources, lack of confidence in accessing wellbeing events/activities and inadequate wellbeing check-in during supervision. Moreover, several barriers were identified including having limited access to information about wellbeing resources, lack of time and fear of stigma/discrimination to access the resources if available.

Following the baseline survey, a document with all the available resources was developed and included in the induction pack for all the resident doctors. A protected wellbeing event was organized with wellbeing activities chosen by the specialty trainee resident doctors. The findings from the survey were also shared with the consultant supervisors in the CPD session. A QI fishbone session was further organized to explore what wellbeing meant to the resident doctors.

Sixteen specialty trainee doctors participated in the follow-up survey. The findings showed improvement in awareness about the resources available to them and confidence in accessing these resources. There was a significant improvement in their perception regarding wellbeing check at workplace (baseline 53.3%, follow up 81.3%). However, there were still concerns around stigma, fear of discrimination that need further exploration and intervention.

**Conclusion:** This project has demonstrated improvement in the specialty trainee resident doctors’ perception about their wellbeing support. However, there are several challenges in terms of sustaining these positive changes and fostering a positive culture of help seeking by mitigating stigma and fear of discrimination.

